# Tree shrew, a potential animal model for hepatitis C, supports the infection and replication of HCV *in vitro* and *in vivo*

**DOI:** 10.1099/jgv.0.000869

**Published:** 2017-07-31

**Authors:** Yue Feng, Yue-Mei Feng, Caixia Lu, Yuanyuan Han, Li Liu, Xiaomei Sun, Jiejie Dai, Xueshan Xia

**Affiliations:** ^1^​ Faculty of Life Science and Technology, Yunnan Provincial Center for Molecular Medicine, Kunming University of Science and Technology, Kunming, Yunnan 650500, PR China; ^2^​ Academy of Public Health, Kunming Medical University, Kunming, Yunnan 650500, PR China; ^3^​ Institute of Medical Biology, Chinese Academy of Medical Sciences and Peking Union Medical College, Yunnan 650118, PR China

**Keywords:** Tree shrew, hepatitis C virus, infection, *in vitro*, *in vivo*

## Abstract

The tree shrew (*Tupaia belangeri chinensis*), a small animal widely distributed in Southeast Asia and southwest China, has the potential to be developed as an animal model for hepatitis C. To determine the susceptibility of the tree shrew to hepatitis C virus (HCV) infection *in vitro* and *in vivo*, a well-established HCV, produced from the J6/JFH1-Huh7.5.1 culture system, was used to infect cultured primary tupaia hepatocytes (PTHs) and tree shrews. The *in vitro* results showed that HCV genomic RNA and HCV-specific nonstructural protein 5A (NS5A) could be detected in the PTH cell culture from days 3–15 post-infection, although the viral load was lower than that observed in Huh7.5.1 cell culture. The occurrence of five sense mutations [S391A, G397A, L402F and M405T in the hypervariable region 1 (HVR1) of envelope glycoprotein 2 and I2750M in NS5B] suggested that HCV undergoes genetic evolution during culture. Fourteen of the 30 experimental tree shrews (46.7 %) were found to be infected, although the HCV viremia was intermittent in *vivo*. A positive test for HCV RNA in liver tissue provided stronger evidence for HCV infection and replication in tree shrews. The results of an immunohistochemistry assay also demonstrated the presence of four HCV-specific proteins (Core, E2, NS3/4 and NS5A) in the hepatocytes of infected tree shrews. The pathological changes observed in the liver tissue of infected tree shrews could be considered to be representative symptoms of mild hepatitis. These results revealed that the tree shrew can be used as an animal model supporting the infection and replication of HCV *in vitro* and *in vivo*.

## Abbreviations

ALT, alanine aminotransferase; E2, envelope glycoprotein 2; HCV, hepatitis c virus; HVR1, hypervariable region 1; IHC, immunohistochemistry; PTH, primary tupaia hepatocytes; RdRp, reverse-transcription quantitative polymerase chain reaction; RT-nPCR, reverse-transcription nested polymerase chain reaction; RT-qPCR, reverse-transcription quantitative polymerase chain reaction; m.o.i., multiplicity of infection; MTT, a 3-(4,5-dimethylthiazol-2-yl)-2,5-diphenyltetrazolium bromide; NS, nostructural protein.

## Introduction

Hepatitis C virus (HCV) is a positive-sense single-stranded RNA virus of the family *Flaviviridae*. HCV infection can be asymptomatic for 10–20 years, but it eventually leads to liver cirrhosis and hepatocellular carcinoma in the majority of patients [1]. The development of direct-acting antiviral agents, including daclatasvir and sofosbuvir, has changed the treatment strategy for HCV infection dramatically. However, the high price of the drugs and treatment failure owing to virus drug-resistance mutations limit the availability of the newer treatments to patients [2]. Thus, the development of effective HCV vaccines and antiviral therapies is still urgently required.

It is well known that animal models play a crucial role in the development of vaccines and treatments, as well as in the characterization of viral life cycles. With the exception of humans, chimpanzees are the only known organisms that are naturally permissive to HCV infection. Because of the growing ethical constraints, limited availability and high costs associated with chimpanzee studies, other animals have been tested for their ability to support HCV infection [3]. Most of the effort has been directed towards the development of small-animal models for HCV infection. Although T-cell- and B-cell-deficient mice, grafted with human hepatocytes, can robustly support HCV infection, they cannot be used in adaptive immunity studies [4]. The development of genetically humanized mice is in progress, but these animal models only allow specific steps in the HCV life cycle to be studied and support limited or no viral replication [5]. Better understanding of the processes of HCV infection and replication requires the development of a permissive and fully immunocompetent small-animal model.

The tree shrew (*Tupaia belangeri chinensis*) is a small animal that is widely distributed in Southeast Asia and southwest China. It has been documented that tree shrews are susceptible to infection with a wide range of human pathogenic viruses, including hepatitis A, B and D viruses, rotavirus and human herpes simplex virus [6]. Primary tupaia hepatocytes (PTHs) have also been proven to be infected *in vitro* with sera derived from chronic HCV-infected patients [7], and the expression of the major HCV receptor gene in the tree shrew could support the entry of HCV pseudoparticles and replication of cell culture-derived infectious HCV (HCVcc) [8]. The abundance of microRNA-122 and its characteristics also supported the use of this animal as a potential model for HCV infection in our previous study [9]. Furthermore, it was revealed that *in vivo* inoculation with the HCV RNA-positive serum could give rise to short-term viremia and the appearance of anti-HCV IgG in tree shrews; however, the infection rate was extremely low [10]. HCV infection and its pathogenicity for tree shrews were also proved by a Japanese group, although the animal number was limited [11]. Therefore, the infection of tree shrews with HCV *in vivo* or *in vitro* still needs to be confirmed in extended experiments using high-quality animals and a more robust HCV. In this study, we used virions produced in a well-established HCV (J6/JFH1) culture system to infect PTHs *in vitro* and tree shrews *in vitro*. Furthermore, second-generation tree shrews born at a clean animal facility were chosen as experimental subjects to guarantee the HCV infection *in vivo*.

## Results

### HCV infection of primary tupaia hepatocytes

**Fig. 1.**
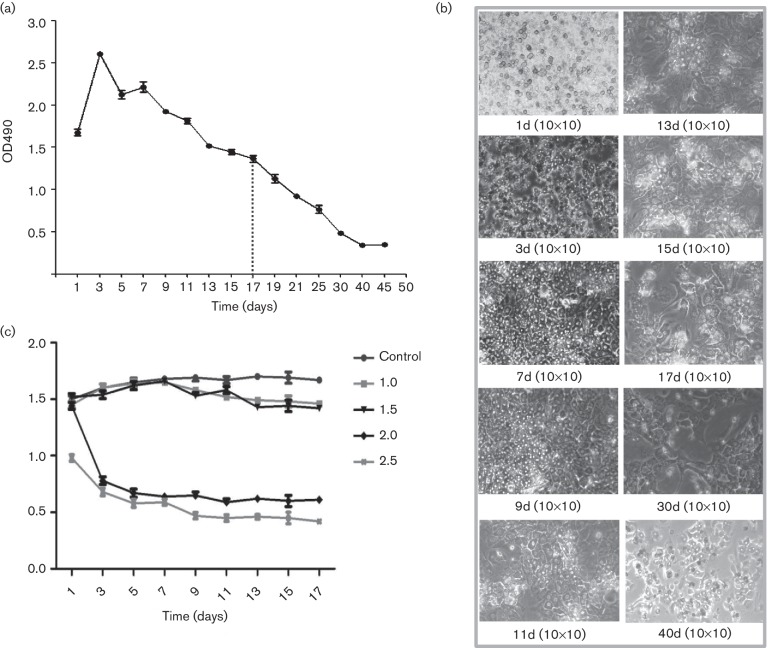
Culture and growth of primary tupaia hepatocytes (PTHs) and effects of HCV infection. Using an MTT assay, the viability of PTHs was determined over a period of more than 30 days, with obvious cell proliferation for 3–9 days in culture (a). The activity and purity of the obtained cells and the cell growth were observed under a microscope (b). HCV infection at an m.o.i. of 2.0 and 2.5 significantly reduced cell viability, while at an m.o.i. of 1.0 and 1.5 there was little effect on the viability of PTH cells (c).

**Fig. 2.**
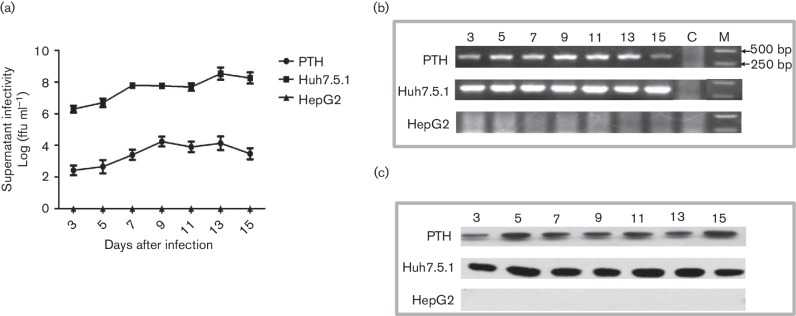
HCV infection and replication in primary tupaia hepatocytes (PTHs). HCVcc was able to infect PTHs, but showed a lower level of replication compared with that in Huh7.5.1 cells (a). HCV RNA (b) and a specific protein (c) were positively detected in the culture supernatants and cell lysates of infected PTHs using RT–nPCR and Western blot, respectively.

PTH cells were used to study the susceptibility of the tree shrew to HCV infection *in vitro*. To determine the optimal time for HCV infection, the viability of the PTHs was evaluated using a 3-(4,5-dimethylthiazol-2-yl)−2,5-diphenyltetrazolium bromide (MTT) assay. As shown in Fig. 1(a, b), a decrease in the cell proliferation of PTHs was observed after 17 days of culture. Therefore, the infection of PTHs with HCV was carried out for 17 days of incubation in subsequent experiments. Afterward, the PTHs were inoculated with HCV at a multiplicity of infection (m.o.i.) of 1.0, 1.5, 2.0 and 2.5 to determine the optimal dose of infection (Fig. 1c). The results showed that cell viability was significantly reduced at an m.o.i. of 2.0 and 2.5, whereas at an m.o.i. of 1.0 and 1.5 there was little effect on the viability of the PTH cells. Thus, to determine whether the chronic-phase virus was acquired by the PTHs of the tree shrew, we inoculated naïve PTH cells with HCV at an m.o.i. of 1.5 for 15 days. Huh7.5.1 and HepG2 cells were used as positive and negative controls. Using the reverse-transcription quantitative and nested polymerase chain reaction (RT-qPCR and RT-nPCR, respectively) methods, we tested for the presence of HCV genomic RNA from 3–15 days post-inoculation every other day in culture supernatants of PTHs, Huh7.5.1 and HepG2 cells. As shown in Fig. 2(a, b), the HCV RNA tests were positive in all the culture supernatants of PTH and Huh7.5.1 at different post-infection times. The results demonstrated a time-dependent increase of HCV RNA after cell incubation with HCVcc, which indicated successful infection of hepatocytes and virus proliferation. However, no virus RNA could be detected in the supernatants of HepG2 cells. Furthermore, Western blotting was used to examine the HCV-specific protein expression in infected PTH, Huh7.5.1 and HepG2 cells. As shown in Fig. 2(c), the HCV nonstructural (NS5A) protein was positively detected in the lysates of PTH and Huh-7.5.1 cells obtained every other day from 3 to 15 days post-inoculation. However, the visualized protein bands from the PTHs were weaker than those from the Huh7.5.1 cells. This is consistent with lower HCV loads in PTH supernatants compared to those in the Huh7.5.1 supernatants (Fig. 2a). The virus-specific protein was not detected in HepG2 cells at any time point post-infection.

**Fig. 3.**
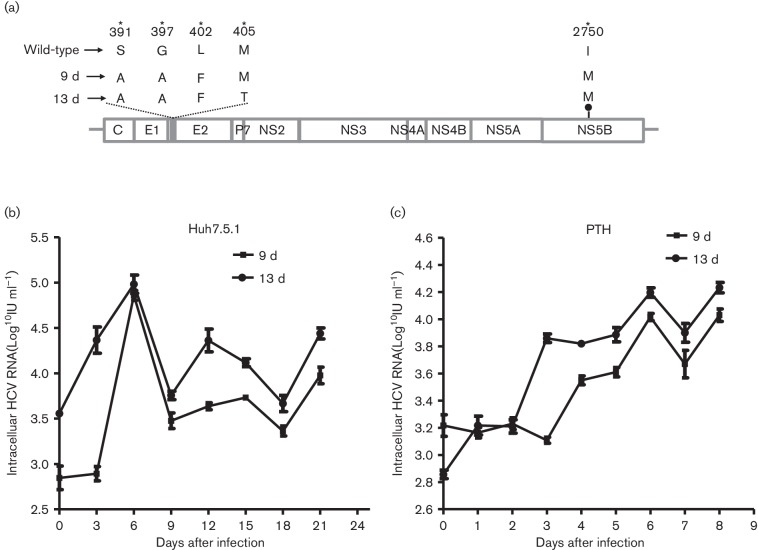
HCV adaptive mutations in PTHs and changes in HCV infectivity. Five sense mutations (S391A, G397A, L402F and M405T in the hypervariable region 1 (HVR1) of E2, and I2750M in NS5B) were revealed in the virus isolated on days 9 and 13 post-infection (a). Then, the media containing mutant viruses from days 9 and 13 were transferred to naïve Huh7.5.1 and PTH cells, and the same mutations were found when infection with the passaged virus was established in these two cell lines (b, c).

To identify the adaptive genetic mutations responsible for HCV infection of PTHs, we sequenced the envelope glycoprotein 2 (E2) and NS5B-encoding regions of the virus collected on days 9 and 13 post-infection. Five sense mutations – S391A, G397A, L402F and M405T in the hypervariable region 1 (HVR1) of E2, and I2750M in NS5B – were revealed in the virus isolated on days 9 and 13 post-infection (Fig. 3a). These results suggested that HCV undergoes genetic evolution during culture. Then, the media from days 9 and 13, containing the mutant virus, infected efficiently and proliferated well in naïve Huh7.5.1 and PTH cells (Fig. 3b, c).

### *In vivo* HCV infection in tree shrews

**Fig. 4.**
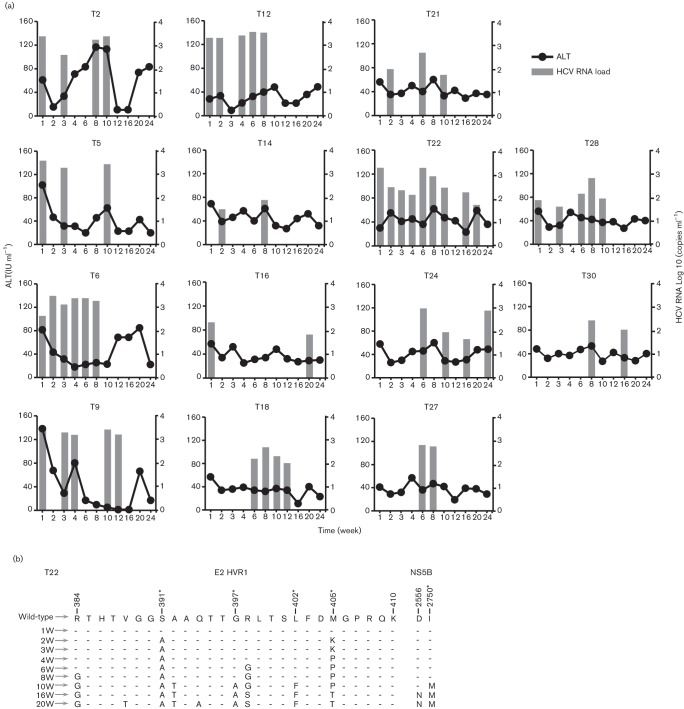
ALT levels and HCV viral load in tree shrews following inoculation with HCV. A total of 14 animals (46.7 %) developed intermittent HCV viremia, as determined by RT–qPCR in the 24 weeks post-inoculation. The ALT levels in these 14 infected animals did not show any obvious changes.

To elucidate the *in vivo* susceptibility of tree shrews to HCV, the virions produced from HCV J6/JFH1-Huh7.5.1 were injected intravenously into the tails of 30 tree shrews, while 10 animals were injected with the supernatant of naïve Huh7.5.1 cells as a negative control. The blood of each animal, anticoagulated with ethylenediaminetetraacetic acid, was collected once a week for 24 weeks to test the HCV viral load and alanine aminotransferase (ALT). Of the 30 tree shrews inoculated with HCV, 14 animals (46.7 %) developed HCV viremia, as determined by RT-qPCR at different times during the 24 week follow-up (Fig. 4a). At the first week post-inoculation, eight animals were positive for HCV RNA. HCV viremia occurred in the other six animals beginning at 2, 6, or 8 weeks post-inoculation. These infected animals showed intermittent viremia over subsequent weeks. HCV RNA could be detected at 2 sampling times in 4 out of the 14 animals with HCV viremia (Fig. 4a). There were five animals that showed HCV viremia at least five times. However, the occurrence of HCV viremia was irregular, and the number of copies of HCV RNA was lower than 10^4^ copies/mL of plasma. This indicates that the tree shrew could support HCV replication *in vivo,* but the HCV replication was ineffective. No HCV infection or replication could be found in the animals of the control group.

To further determine the presence of HCV in the liver, HCV RNA was extracted from surgical liver tissue at 24 weeks post-inoculation. The results of RT and PCR amplification showed that the liver tissue was RNA-positive in all 14 animals with viremia. To evaluate the liver damage caused by HCV infection, ALT levels were measured in the plasma (Tables S1 and S2, available with the online Supplementary Material). The results showed no obvious changes in the ALT levels in the experimental group during the study period, although irregular increases were observed at some time points. To study the adaptive mutations of the virus *in vivo*, we sequenced partial E2 and NS5B derived from sera of tree shrew T22 at different time points, which showed persistent HCV viremia. Compared with the wild-type HCVcc, five mutations, including R384G, S391A, G397A, L402F and M405T, were observed in the HVR-1 of the E2 protein. Two additional substitutions, D2556N and I2750M, were found in the NS5B protein (Fig. 4b).

### Immunohistochemical and pathological changes in liver tissue

**Fig. 5.**
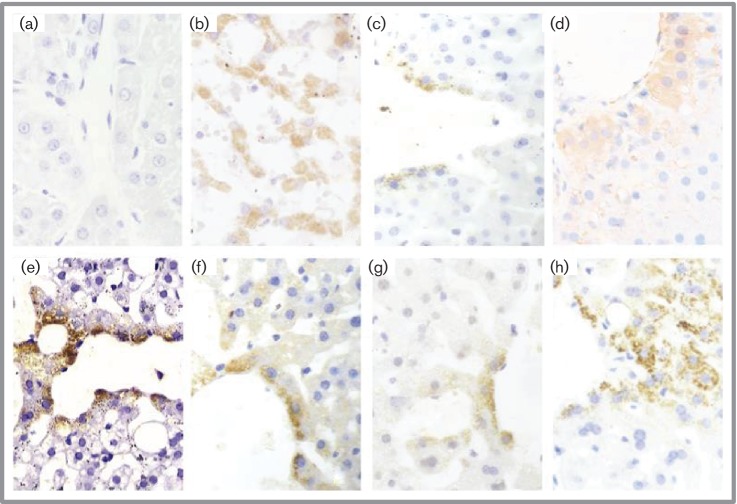
Immunohistochemical detection of HCV-specific proteins using a anti-substance P (SP) method. In the liver tissue of a representative animal (T22), which was infected with Huh7.5.1-produced HCV, E2 (e), core (f), NS5A (g) and NS3/4 (h) proteins were detected in the cytoplasm of the hepatocytes surrounding the central vein of the liver lobule (DAB dark yellow granular cytoplasmic staining, 400×). The same positive NS5A staining pattern is also obvious in the tissue of a hepatitis C patient (b) and a tree shrew infected with a clinical HCV strain (c). The red APE colour (d) also shows the presence of the NS5A protein in the hepatocytes of T22. By comparison, no NS5A staining was observed in the liver tissue of the negative control tree shrew (a).

The HCV core, E2, NS3–NS4 and NS5A proteins were detected in the liver tissue of infected animals with specific monoclonal antibodies using an indirect immunohistochemical assay. Differences in the presence of the proteins in the liver could be detected among the animals. In particular, the presence of the four proteins was confirmed in the liver tissue of the T22 animal, which was found to have more persistent HCV viremia. Dark yellow staining showed the dispersed distribution of the HCV core (Fig. 5f), E2 (Fig. 5e), NS5A (Fig. 5g) and NS3/4 (Fig. 5h) proteins in the cytoplasm of the liver cells that surrounded the central vein of the liver lobule (Fig. 5). An APE red colour (Fig. 5d) also indicated the presence of the NS5A protein in the hepatocytes of T22 infected with the Huh7.5.1 cell-derived HCV. More obvious staining in the near-vein hepatocytes indicated that HCV proliferated from near- to far-vein cells because we used the most efficient infection route, vein injection. The HCV NS5A protein was also found to be present in liver cells from patient plasma HCV-infected animals (Fig. 5c) and a hepatitis C patient (Fig. 5b). By contrast, no HCV NS5A protein could be found in the naïve tree shrews without HCV infection (Fig. 5a).

**Fig. 6.**
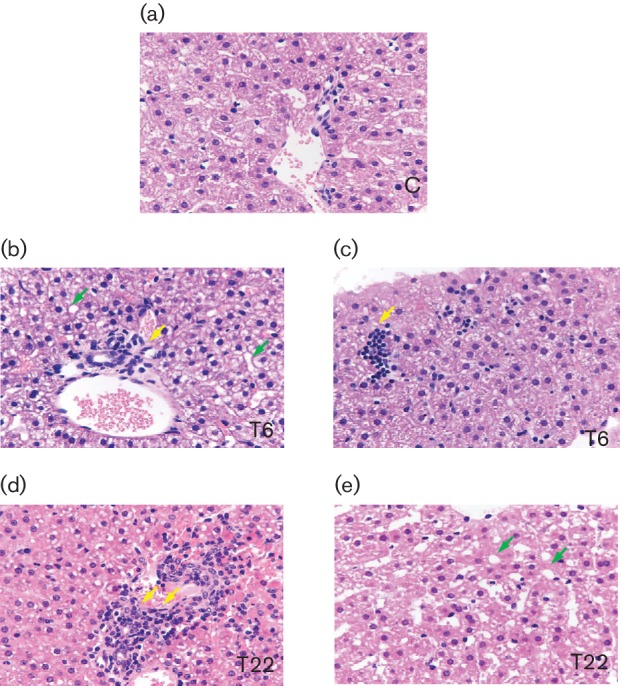
Micrographs of liver specimens stained with H and E (40×). Liver tissue was collected from HCV-infected tree shrews, with T6 and T22 as representative animals, 24 weeks post-inoculation. Liver specimens from uninfected animals (control group), matched to infected animals, were also obtained. The HCV-infected tupaia livers harboured infiltrating lymphocytes (yellow arrowheads) and showed microvesicular fat accumulation (green arrowheads).

Pathologically, various degrees of microvesicular fat accumulation and vacuolar degeneration could be observed in the hepatocytes of infected animals. An obvious hepatic edema, the infiltration of a few phlogistic cells into the portal area, and spotty or focal necrosis could be found in the liver tissue of the T6 animal (Fig. 6). Steatosis was observed in some hepatocytes, while lymphocyte proliferation was observed in the hepatic sinusoid, and lymphocytic infiltration was observed in the portal area and between biliary epithelial cells (Fig. 6). By contrast, the structure of the hepatocytes in the control animals was normal under a light microscope (Fig. 6). Taken together, these pathological changes in the liver tissue of infected tree shrews could be considered to be representative symptoms of mild hepatitis.

## Discussion

*In vitro* HCV culture systems are essential tools to investigate the virus proliferation and pathogenic mechanism, screen antiviral compounds and develop preventive vaccines [12]. Before 2005, several efficient HCV culture systems had been established [13–15]. An HCV genotype 2a replicon (JFH), which efficiently replicates in human hepatoma Huh7 cells without inducing adaptive mutations, was used to construct transfection plasmids in this culture system [14]. Furthermore, the more robust infectivity of J6/JFH1 chimeric HCVcc was proven based on its long-term infection in chimpanzees and mice containing human liver grafts [16]. Since then, J6/JFH1 HCVcc has been used widely in research on HCV cell entry and receptor binding, and for the establishment of cell or animal models [12]. Although several types of primary hepatocytes could be infected successfully by serum-derived HCV, the use of these systems is still limited, owing to a low level of replication [17]. In this study, a well-established HCV produced in the J6/JFH1-Huh7.5.1 culture system was used to infect cultured PTHs and tree shrews. The *in vitro* results showed that HCV genomic RNA and an HCV-specific protein (NS5A) could be detected in the PTH cell culture from 3–15 days post-infection, in agreement with the report that indicated that PTHs are susceptible to HCV infection [7]. Further, we found that the cell culture supernatants derived from 9- and 13-day PTH culture were able to infect naïve Huh7.5.1 and PTH cells efficiently, indicating the production of an infectious virus in PTHs, in agreement with a previous report [7].

As early as 1978, tree shrews had been proven to be experimentally infected with herpes simplex virus [18]. Since then, the susceptibility of this animal to hepatitis viruses (A to E), rotavirus, enterovirus 71 and adenovirus has been reported [6]. The possibility of using the tree shrew as an animal model for viral diseases has also been supported by the characteristics of its genomic sequence and its unique immune system, e.g. the lack of RNA helicase RIG-I [19, 20]. For the development of a hepatitis C animal model, *in vivo* infection of tree shrews has been proven repeatedly with patient plasma-derived HCV, although the infection rates were low and inconsistent [7, 10, 21]. Using cell culture-produced HCV supernatants, a Japanese group established a tree shrew model for chronic hepatitis C, with obvious representation of cirrhosis [11]. In the present study, 14 of the 30 experimental tree shrews (46.7 %) were infected with HCV supernatants produced from J6/JFH1-transfected Huh7.5.1 cells. Although the HCV viremia was inconsistent, HCV RNA could be detected in liver tissue, indicating that HCV persists in tupaias, which provided stronger evidence for HCV infection and replication in tree shrews. Impressively, the results of an immunohistochemistry (IHC) assay also demonstrated the presence of four HCV-specific proteins (core, E2, NS3/4 and NS5A) in the hepatocytes of infected tree shrews. This indicates that HCV protein translation, a critical step in the life cycle, occurs in the cells. Interestingly, the irregular or unapparent ALT increase [22] and HCV protein cytoplasmic staining pattern [23] described in hepatitis C patients were also found in these infected tree shrews. All of these results provided more evidence for the suitability of the tree shrew as an HCV animal model [11]. However, we only found pathological changes consistent with mild hepatitis in the two animals T6 and T22, but no apparent cirrhosis, which has been demonstrated for the documented chronic HCV-infected tree shrew model. In contrast, only mild hepatic steatosis was observed in the remaining 12 animals with HCV viremia (data not shown). This may be due to the fact that the viremia was intermittent and accompanied by relatively low HCV titres. Furthermore, HCV caused liver damage that worsened in a time-dependent manner. Therefore, it will be necessary to monitor disease progression in HCV-infected tree shrews for a long time, perhaps for 3 or more years.

In addition to using the tree shrew as a hepatitis C animal model, *in vitro* PTHs provide a potentially useful tool for research on HCV infection in primary hepatocytes and for the screening of anti-HCV compounds. This would also help us to obtain more evidence for the establishment of an animal model. A two-step collagenase perfusion procedure has been used for the isolation of primary hepatocytes from humans, rats, mice, swine, etc. [24]. Using this method for the isolation of PTHs, with subsequent culture in Williams' E medium, the activity and purity of the obtained cells was approximately 70 %, based on MTT detection and microscopic observation. Furthermore, the lifespan of the cultured PTHs was more than 30 days, with obvious cell proliferation for a 3–9 day culture period. These PTH features could ensure HCV infection and virus replication. In the last decade, PTHs have been widely used for the establishment of hepatitis B virus (HBV) and HCV infection models *in vitro* [25]. In addition to HBV infection of PTHs, HCV has been documented to infect and replicate in cultured PTHs as a potential model for HCV infection, which led to the selection of virus quasispecies, the induction of interferon-stimulated genes and nuclear factor-kappa B nuclear translocation [7, 26]. Here, an HCV supernatant produced from the J6/JFH1-Huh7.5.1 culture system was used to infect cultured PTHs. The support of HCV infection and replication by PTHs was proven by qualitative and quantitative RNA detection, as well as by HCV-specific protein detection using Western blotting. However, the HCV replication was less efficient than that in Huh7.5.1 cells. This is consistent with the data from our previous studies, which showed lower levels of HCV cell entry and induction of major receptors, including clusters of differentiation 81 (CD81), scavenger receptor class B member 1 (SRB1), claudin-1 and occludin, in PTHs than in human cells [8]. Thus, further research is necessary to provide evidence for the feasibility of the tree shrew as an animal model for hepatitis C.

In the present study, we also evaluated the adapted mutations of HCV HVR1 and NS5B *in vitro* and *in vivo*. HVR1 evolution generates HCV variants that have advantages for maintaining persistent HCV infection and viral fitness under the specific conditions of the host infection [27]. This region best reflects the complexity and diversity of HCV, and has been also studied extensively in infected patients and chimpanzees [28, 29]. Furthermore, it was previous reported that some mutations generated in the NS5B region during chronic infection may lead to a major increase in viral RNA replication [30]. Interestingly, our results revealed that five sense mutations – S391A, G397A, L402F and M405T in HVR1, and I2750M in NS5B – were not only observed *in vitro*, but also *in vivo*, indicating that the HCV strains in the tree shrew may be undergoing RNA replication and quasispecies evolution. These data are consistent with longitudinal analyses of H77 quasispecies evolution in humans and chimpanzees revealing the time-dependent development of new mutations [7, 31, 32]. However, whether the mutation I2750M in NS5B can result in a major increase in viral RNA replication or not, which is necessary to study the RdRp activity *in vitro* using a site-directed mutation method in the future.

Tree shrews, which are non-rodent, primate-like small animals, are phylogenetically close to primates. Other than the chimpanzee, the tree shrew is the only animal in nature that has been shown to be infected by HCV thus far. However, the wild nature of the animal and the difficulty of breeding it in a laboratory setting have hindered its widespread use. In our laboratory, techniques and facilities for the maintenance of wild-captured tree shrews have been established. Using the breeding technique we established in-house, we can obtain third generation laboratory-born babies. An environment that is free of specific pathogens can ensure strict experimental conditions for this animal.

## Methods

### Animals

Second filial generation tree shrews with body weights between 80 and 110 g (20 males and 20 females, 6 months old) were used in this study. All of the tree shrews were kept individually at the animal facilities of Kunming University of Science and Technology at a temperature of 25±1 °C with 50 % relative humidity. The animals were documented to be free of specific pathogens, particularly HBV and HCV. All procedures involving animals were approved by the Animal Care and Use Committee of Kunming University of Science and Technology, China.

### Culture of primary tupaia hepatocytes and HCV infection

Primary hepatocytes of tupaia were isolated from adult tree shrews by a two-step collagenase perfusion procedure [33]. Freshly isolated hepatocytes were seeded at 1×10^5^ cells ml^−1^ in Williams' E medium supplemented with penicillin (100 U ml^−1^), streptomycin (100 µg ml^−1^), bovine insulin (5 mg l^−1^), 2 % dimethyl sulfoxide and 10 % foetal calf serum. The viability of the cultured PTH cells was examined for 45 days using an MTT assay. Cultured PTHs were infected with cell culture-grown HCVcc (J6/JFH1) produced from transfected Huh7.5.1 cells [13]. Huh7.5.1 and HepG2 cells were used as positive and negative controls. The infection medium was removed after 8 h, and the wells were washed with 1 ml of culture medium eight times. Subsequently, 500 µl of fresh medium was added to continue the culturing for 17 days, with a medium change every 48 h. The culture supernatants were collected on days 1, 3, 5, 7, 9, 11, 13, 15 and 17 post-infection for HCV RNA load determination using a commercial qRT–PCR kit (PG Biotec Co.).

### *In vivo* infection of tree shrews with HCV

Infectious HCVcc (J6/JFH1) was used for the infection of tree shrews. For the study, 40 tree shrews were chosen and randomly divided into 2 groups, an experimental group (*n*=30; 15 males and 15 females) and a negative control group (*n*=10; 5 males and 5 females). Inoculation was performed intravenously in the tail at 1×10^7^ genome equivalents per animal with reconstituted virions derived from the J6/JFH1 inoculation. The negative control animals were inoculated with the cell culture medium of naïve Huh7.5.1 cells instead of the virus, in the same way as the experimental group. At weeks 1, 2, 3, 4, 6, 8, 10, 12, 16, 19 and 22 post-inoculation, blood was drawn from the tail vein of fasted tree shrews to determine the ALT using an ALT reagent kit (Rongsheng-Bio). Plasma was separated and used for the detection of the HCV load. The experimental animals were sacrificed after the last blood draw, and the liver of each animal was collected for hematoxylin and eosin (H and E) staining and IHC analysis.

### HCV amplification and determination of adaptive mutations

Total RNA was extracted from cell-culture supernatants and the plasmas and livers of animals using Trizol reagent (Invitrogen) according to the manufacturer's instructions. Owing to the high degree of conservation and amplification efficiency of the 5′ non-coding region (5′-NCR), this region was used to evaluate the HCV infection rate. The hypervariable region 1 (HVR1) in the E2 protein and RNA-dependent RNA polymerase (RdRp) in NS5B were used to determine the potential adaptive mutations *in vitro or in vivo*, as the genetic evolution of HCV infection has been characterized in detail in humans and chimpanzees. RT-nPCR was performed using these primers and optimal PCR amplification conditions (Table S3). The first PCR reaction was performed using one-step reverse transcription PCR (Takara) and the second was performed using 2× high-fidelity *Taq* PCR MasterMix (Tiangen Biotech Co.). The PCR products were analysed by agarose gel electrophoresis, and the positive PCR samples were purified using the PCR product gel extraction kit (Tiangen Biotech Co.) and then sequenced by Invitrogen. The obtained sequences were compared with those in GenBank using blast to confirm their identity with HCV HVR1 and RdRp, and to search for adaptive mutations compared with the wild-type HCVcc derived from J6/JFH1.

### Western blotting

Cell lysates of PTHs were prepared using a protein lysis buffer (Beyotime Biotech Co.). The protein concentration was determined using a protein assay reagent (Bio-Rad). A total of 30 µg of protein was separated by 15 % sodium dodecyl sulfate polyacrylamide gel electrophoresis and transferred to a polyvinylidene difluoride membrane (Roche Diagnostics). The membrane was blocked with 5 % (w/v) skimmed milk for 2 h at room temperature and then incubated with primary antibodies against NS5A (Cell Signaling Technology) and β-actin (EnoGene Biotech Co.) overnight at 4 °C, followed by incubation with peroxidase-conjugated anti-mouse and anti-rat or anti-rabbit IgG for 1 h at room temperature (KPL, Inc.). The immunoreactive epitopes were visualized using an enhanced chemiluminescence Western blot detection kit (Millipore).

### IHC and histological analysis

Liver tissues were collected from anaesthetized animals 24 weeks post-inoculation. HCV antigens in the surgical liver tissue of infected tree shrews were detected with a two-step indirect immunostaining procedure using an ultra-sensitive anti-substance P (SP) assay (Maixin-Bio Ltd.). In brief, 5 % formalin-fixed liver tissue was embedded in paraffin and then cut into 3 µm slices. After hydration and antigen retrieval, anti-core, E2, NS3–NS4 and NS5A monoclonal antibodies, and a biotin-conjugated secondary antibody, were reacted, and the slices were subsequently stained with streptavidin–peroxidase. Finally, a coloured reaction was performed using diaminobenzidine (DAB) or 3-amino-9-ethylcarbazole (AEC). For the DAB-coloured reaction, sections were counterstained with hemalum, dehydrated in graded alcohol, cleared in xylol and mounted with Eukitt mounting medium. The dark yellow (DAB)- or red (AEC)-stained HCV antigens and their cellular localization were visualized by microscopy. Hepatitis C patient tissue and naïve tree shrew tissue were used as positive and negative controls, respectively. At the sampling time, liver tissue was fixed in 10 % neutral buffered formalin, embedded in paraffin, sectioned and stained with H and E. All histological staining was performed in accordance with conventional procedures. Histological changes were observed by microscopy.

## Supplementary Data

344 Supplementary File 1
