# Supplementary material for: Tree shrew, a potential animal model for hepatitis C, supports the infection and replication of HCV in vitro and in vivo
Source: J Gen Virol. 2017 Jul 31;98(8):2069–78. doi: 10.1099/jgv.0.000869 (PMC5656785; doi:10.1099/jgv.0.000869)
Supplement: Supplementary File 1 [file jgv-98-2069-s001.pdf]

Table S1：Evolution of ALT levels in infected Tupaia with HCV during 24 weeks

**Weeks**	**ATL value (U) of the infection samples**																													
	**T1**	**T2**	**T3**	**T4**	**T5**	**T6**	**T7**	**T8**	**T9**	**T10**	**T11**	**T12**	**T13**	**T14**	**T15**	**T16**	**T17**	**T18**	**T19**	**T20**	**T21**	**T22**	**T23**	**T24**	**T25**	**T26**	**T27**	**T28**	**T29**	**T30**
1	27	***76***	91	78	***102***	***81***	80	56	***138***	46	38	***28***	85	***69***	84	***57***	56	***57***	64	57	***56***	***30***	79	***58***	33	33	***41***	***56***	46	48
2	47	***19***	88	75	***47***	***43***	79	38	***67***	42	29	***34***	76	***39***	84	***34***	38	***34***	57	55	***35***	***55***	66	***26***	32	32	***29***	***29***	75	32
3	20	***42***	64	60	***32***	***32***	69	29	***29***	49	15	***9***	66	***46***	76	***52***	29	***36***	76	32	***37***	***41***	62	***30***	96	96	***32***	***32***	49	40
4	91	***89***	62	52	***31***	***18***	59	27	***80***	49	57	***22***	63	***57***	57	***25***	27	***39***	66	32	***50***	***45***	58	***45***	46	46	***57***	***54***	64	37
6	43	***104***	54	49	***20***	***22***	57	25	***17***	32	32	***33***	62	***40***	50	***31***	25	***34***	82	29	***40***	***36***	45	***46***	56	56	***36***	***45***	57	47
8	67	***146***	44	48	***46***	***25***	44	22	***10***	55	46	***40***	57	***61***	40	***34***	22	***32***	73	26	***60***	***62***	40	***60***	42	42	***47***	***42***	57	53
10	40	***142***	40	42	***63***	***23***	34	20	***5***	75	43	***48***	43	***32***	31	***48***	20	***37***	81	87	***33***	***47***	26	***29***	51	51	***42***	***37***	76	27
12	49	***13***	32	42	***23***	***69***	31	17	***1***	28	50	***21***	41	***27***	25	***32***	17	***34***	72	74	***42***	***42***	25	***27***	49	49	***19***	***38***	66	42
16	34	***13***	31	35	***23***	***69***	18	12	***1***	67	22	***21***	36	***44***	25	***27***	12	***11***	63	54	***29***	***23***	17	***31***	82	62	***39***	***27***	63	33
20	43	***92***	30	32	***43***	***85***	18	34	***66***	43	30	***36***	31	***52***	14	***29***	34	***40***	28	31	***37***	***59***	94	***48***	49	82	***38***	***43***	82	28
24	61	***104***	65	64	***20***	***22***	36	29	***17***	54	43	***49***	34	***32***	31	***30***	49	***23***	46	30	***35***	***36***	58	***49***	62	55	***29***	***40***	73	40

The data of 14 animals with HCV viremia were marked in blue.

Table S2： Evolution of ALT levels in uninfected *Tupaia* during 24 weeks

Weeks	ALT value (U) of the noninfection samples									
	**C1**	**C2**	**C3**	**C4**	**C5**	**C6**	**C7**	**C8**	**C9**	**C10**
1	63	36	43	85	18	46	38	71	56	38
2	21	34	31	76	30	42	29	63	20	29
3	32	35	52	66	40	49	15	56	37	15
4	51	33	41	63	71	49	57	48	31	57
6	8	2	6	62	22	32	32	44	27	32
8	19	19	43	57	19	55	46	33	34	46
10	2	5	64	43	21	67	18	24	56	43
12	10	5	19	41	13	43	17	23	56	18
16	10	5	19	36	16	54	20	5	51	17
20	72	28	42	31	66	62	21	43	82	20
24	8	2	6	24	44	53	13	57	33	21

Table S3. PCR primers and amplification conditions

Gene	Primer name	Position*	PCR Products Size (bp)	HCV primer sequence	Amplification conditions
5'NCR	5UTRF01	37-62	359	5-CACTCCCCTGTGAGGAACTACTGTCT-3	50°C 30min, 95°C 3 min, 95°C 30 sec 50°C 30
	5UTRR01	430-454		5-CGGCAACAAGTATACTCCGCCAACG-3	sec 72°C 0:50 min (34×), 72°C 5 min
	5UTRF02	64-85		5-CACGCAGAAAGCGCCTAGCCAT-3	95°C 3 min, 95°C 30 sec 55°C 30 sec 72°C 0:40
	5UTRR02	400-422		5-CGCCGCCCGGGAACTTAACGTCT-3	min (29×), 72°C 5 min
E2	E2F01	1209-1232	688	5-TGTTCATTGTCTCGCCGCAGCACC-3	50°C 30min, 95°C 3 min, 95°C 30 sec 55°C 30
	E2R01	1997-2022		5-CCAGAAGAGTTCATCCACGTGCAGCC-3	sec 72°C 1:40 min (34×), 72°C 5 min
	E2F02	1293-1319		5-TGGCATGGGACATGATGATGAACTGGT-3	95°C 3 min, 95°C 30 sec 58°C 30 sec 72°C 1:10
	E2R02	1952-1980		5-GGTGGTCGAGTGCTGTTCAATAGGAAGAC-3	min (29×), 72°C 5 min
NS5B-F1	F1NS5BF01	7551-7578	1069	5-TGGAGTCTGATCAGGTAGAGCTTCAACC-3	50°C 30min, 95°C 3 min, 95°C 30 sec 50°C 30
	F1NS5BR01	8696-8722		5-ATCACCAGGAGGGGCAGAGTACCTGGT-3	sec 72°C 2:30 min (34×), 72°C 5 min
	F1NS5BF02	7620-7646		5-GGTCTTGGTCTACTTGCTCCGAGGAGG-3	95°C 3 min, 95°C 30 sec 60°C 30 sec 72°C 2:30
	F1NS5BR02	8665-8688		5-TCCGTGAAGGCTCTCAGGTTCCGC-3	min (29×), 72°C 5 min
NS5B-F2	F2NS5BF01	8515-8539	895	5-GGTGCTAACCACTAGCATGGGTAAC-3	50°C 30min, 95°C 3 min, 95°C 30 sec 52°C 30
	F2NS5BR01	9454-9481		5- GGAACAGTTAGCTATGGAGTGTACCTAG-3	sec 72°C 2:30 min (34×), 72°C 5 min
	F2NS5BF02	8565-8592		5-TAGCGGCCTGCAAGGCTGCGGGGATAGT-3	95°C 3 min, 95°C 30 sec 60°C 30 sec 72°C 2:30
	F2NS5BR02	9433-9459		5-ACCTAGTGTGTGCCGCTCTACCGAGCG-3	min (29×), 72°C 5 min

*Nucleotide positions are numbered as for AB047639.
